# Phenotypic plasticity in the range-margin population of the lycaenid butterfly *Zizeeria maha*

**DOI:** 10.1186/1471-2148-10-252

**Published:** 2010-08-19

**Authors:** Joji M Otaki, Atsuki Hiyama, Masaki Iwata, Tadashi Kudo

**Affiliations:** 1 The BCPH Unit of Molecular Physiology, Department of Chemistry, Biology and Marine Science, Faculty of Science, University of the Ryukyus, 1 Senbaru, Nishihara, Okinawa 903-0213, Japan; 2 Tsugaru Insect Club, 4-13-1 Aoyama, Hirosaki, Aomori 036-8062, Japan

## Abstract

**Background:**

Many butterfly species have been experiencing the northward range expansion and physiological adaptation, probably due to climate warming. Here, we document an extraordinary field case of a species of lycaenid butterfly, *Zizeeria maha*, for which plastic phenotypes of wing color-patterns were revealed at the population level in the course of range expansion. Furthermore, we examined whether this outbreak of phenotypic changes was able to be reproduced in a laboratory.

**Results:**

In the recently expanded northern range margins of this species, more than 10% of the *Z. maha *population exhibited characteristic color-pattern modifications on the ventral wings for three years. We physiologically reproduced similar phenotypes by an artificial cold-shock treatment of a normal southern population, and furthermore, we genetically reproduced a similar phenotype after selective breeding of a normal population for ten generations, demonstrating that the cold-shock-induced phenotype was heritable and partially assimilated genetically in the breeding line. Similar genetic process might have occurred in the previous and recent range-margin populations as well. Relatively minor modifications expressed in the tenth generation of the breeding line together with other data suggest a role of founder effect in this field case.

**Conclusions:**

Our results support the notion that the outbreak of the modified phenotypes in the recent range-margin population was primed by the revelation of plastic phenotypes in response to temperature stress and by the subsequent genetic process in the previous range-margin population, followed by migration and temporal establishment of genetically unstable founders in the recent range margins. This case presents not only an evolutionary role of phenotypic plasticity in the field but also a novel evolutionary aspect of range expansion at the species level.

## Background

Mostly due to climate warming and its associated environmental changes, recent studies have revealed the expansion of the geographical distributions of several species, including many European [1-4], American [5], and Japanese [6-8] butterflies. Although habitat associations are generally constrained at range margins, physiological adaptation was observed in British butterflies in the range expansion process [1,3,4]. In these cases, the habitat variations that certain butterfly species can colonize increased over time [1,3,4]. Such changes are well within the range of normal physiological adaptation to different environmental conditions.

**Figure 1:**
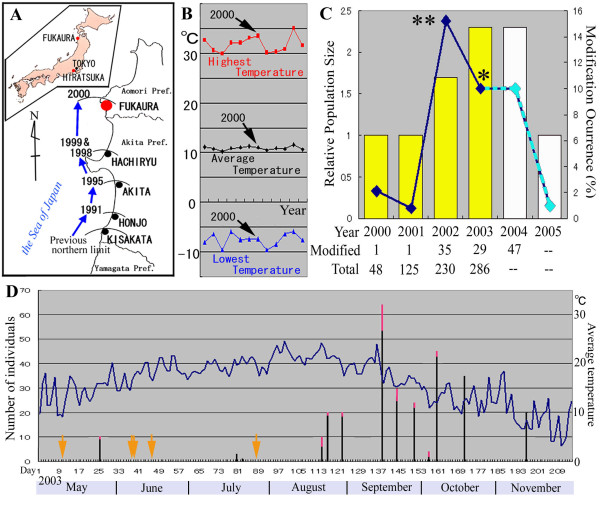
**Field work on *Z. maha *in Fukaura, Aomori Prefecture, Japan**. (**A**) Range expansion of *Z. maha *to the north in Honshu Mainland, Japan. Inset shows the locations of Fukaura, Hiratsuka, and Tokyo in the Japanese Islands. (**B**) Temperature dynamics of Fukaura from 1994 to 2005. Highest (shown in red), average (black), and lowest (blue) temperature records for a given year are indicated. The highest temperature in Fukaura had steadily increased year by year since 1996 until 2000. In 2001, the highest and lowest temperature suddenly dropped. (**C**) Estimated percentages of the color-pattern modified individuals among the total population of *Z. maha *in Fukaura (line graph). Relative population size is indicated as bar graph. Asterisks indicate a possible psychological bias. That is, the 2002 data (indicated by double asterisks) may be an overestimate, and the 2003 data (a single asterisk) may be an underestimate. Similarly, for 2004 and 2005, the modification occurrence is connected with broken lines (instead of continuous lines) and the relative population size is indicated by white bars (instead of yellow bars) to indicate the incompleteness of data records for these years. (**D**) Daily fluctuations of average temperature and the daily occurrence of the modified individuals in 2003. Red portion in a bar indicates the number of modified individuals that were caught, which are mostly from the late August to the early October when temperature is relatively high. Arrows indicate days when the field work was carried out without any *Z. maha *individual confirmed.

The Japanese pale grass blue butterfly, *Zizeeria maha*, is a species of Japanese lycaenid butterfly with an expanding distribution range probably due to climate warming. We have previously reported that the distribution of *Z. maha *had been expanding northward since 1991 and it reached Fukaura, Aomori Prefecture, the northernmost part of Honshu Mainland, Japan, in 2000 (Fig. 1A) [7,8]. Because butterfly distribution records in Japan are thoroughly described by professional and amateur lepidopterists, and because butterflies of the Aomori and Akita region of Japan are indeed thoroughly described [9,10], it is reasonable to think that the recent collections of *Z. maha *in Fukaura and other areas are associated with a northward range expansion of the species and are not artifacts of previously unknown populations. Moreover, similar northward range expansion was found not only in *Z. maha*, but also in other butterflies such as *Papilio protenor*, *Papilio memnon*, and *Junonia almana *[6-8], supporting the notion that the northern range expansion of *Z. maha *is a real phenomenon and is highly likely to be due to the warming climate.

We here report that the northern expansion of *Z. maha *was accompanied by an extraordinary phenomenon: the outbreak of color-pattern modifications. In contrast to most species, which exhibited normal physiological adaptation to new environmental conditions, *Z. maha *individuals at the recently expanded northern range margins, Fukaura, Japan, showed various color-pattern changes on their wings with no other obvious aberrations. In this paper, we document an observation of phenotypic diversification of *Z. maha *in the field. We reproduced the modified phenotypes physiologically as well as genetically in our laboratory. Based on these data, we discuss possible mechanisms of this outbreak and the importance of phenotypic plasticity in butterfly wing color-pattern evolution.

## Results

### Outbreak of the modified forms in Fukaura

*Zizeeria maha *is generally considered to be vulnerable to low temperature conditions [11,12]. Hence, the recently reported northward range expansion of this species [7,8] led us to investigate recent temperature dynamics in Fukaura. The highest temperatures in Fukaura had steadily increased year by year since 1996 until 2000 (Fig. 1B), which probably encouraged the range expansion of *Z. maha *to the north. On the other hand, a large amount of snowfall in Fukaura did not discourage this range expansion (not shown).

**Figure 2:**
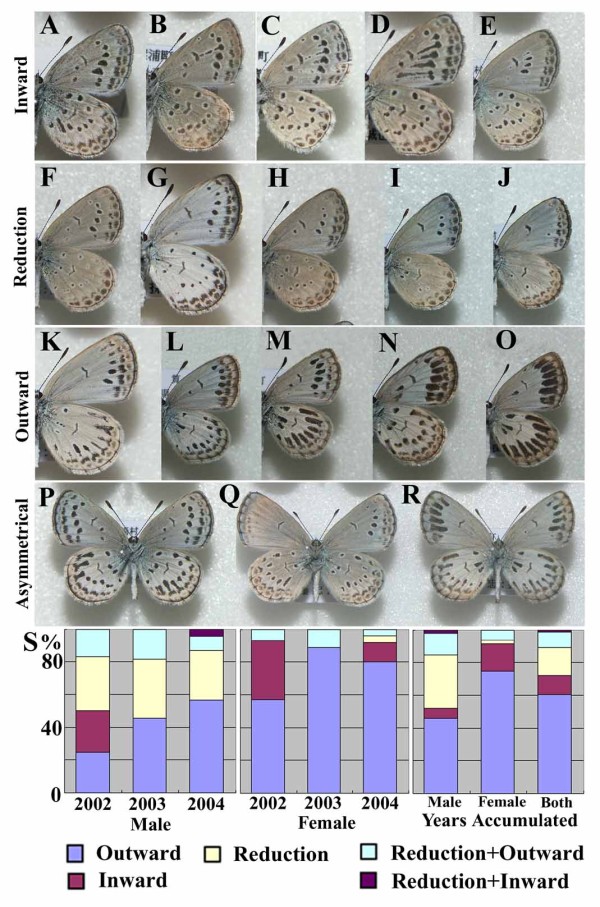
**Modified individuals obtained from the Fukaura area**. Various degrees of modifications were observed, but spot elongation (both inward and outward elongation) and spot reduction (or disappearance) were key features. (**A-E**) Individuals of the inward-type modifications. (**F-J**) Individuals of the reduction-type modifications. (**K-O**) Individuals of the outward-type modifications. (**P-R**) Individuals of asymmetrical modifications. (**S**) Modification-type profiles of *Z. maha *caught in the Fukaura area in 2002, 2003, and 2004. The outward type increased from 2002 to 2004 especially in male, but the reduction type seems to be almost constant in male.

What is quite unusual and interesting in this case is that a number of *Z. maha *individuals in Fukaura exhibited unique color-pattern modifications on the ventral side of their wings (Fig. 2). In 2000 and 2001, the proportion of these modified individuals was estimated to be less than 2% (only a few modified individuals were caught), all of which showed slight modifications (Fig. 1C). In 2002, the percentage of the modified individuals increased to 15% (35 modified individuals out of 230 caught), including individuals with slight modifications. In 2003 and 2004, the percentage of modified individuals was estimated to be similar to the amount seen in 2002, many of which exhibited small degree of modifications. However, despite excluding the individuals with small degree of modifications, the percentage of modified individuals reached 9.7% (29 severely modified individuals out of 300 caught) in 2003.

During this period, the relative population size also increased, albeit more slowly than the percentage of modified individuals. In 2005, the percentage of the modified individuals suddenly decreased. The occurrence of modified individuals from 2000 to 2005 correlates with the overall yearly population size (Pearson correlation coefficient 0.78). These figures, although rough, readily indicate that population-level modifications of *Z. maha *occurred in Fukaura in 2002, 2003, and 2004.

The color-pattern changes appeared to occur mostly from late August to early October in 2003 (Fig. 1D). During this period, the average temperature peaked in Fukaura. Similar results were obtained in 2002 and 2004 (not shown). However, high incidence of the modified individuals in summer is likely to be a simple reflection of large seasonal population size in summer (see below).

### Classification of the Fukaura individuals: three modification types

The color-pattern modifications found in the Fukaura population can largely be classified into three types (Fig. 2A-O). The first type, "the inward type", shows black spots elongating toward the wing base (Fig. 2A-E). The second type, "the reduction type", shows simple size reduction or loss of spots (Fig. 2F-J). The third type, "the outward type", exhibits black spots elongating toward the outer margin (Fig. 2K-O). Most individuals exhibit symmetrical modifications on both right and left wings in all three modification types. Some exceptional individuals, however, exhibit asymmetrical spot modifications (Fig. 2P-R).

These three modification types (*i.e.*, the inward, reduction, and outward types) were generally found in both sexes in the years 2002, 2003, and 2004, but their proportion among the total number of the modified individuals varied between sexes and among years (Fig. 2S). In both sexes, but especially in females, the majority of the modified individuals were of the outward type. In males, the percentage of the outward type increased stepwise from 2002 to 2004. In males but not in females, the reduction type also occupied a significant proportion of the modified population, and its proportion was nearly constant over three years. The inward type was observed mostly in 2002 in both sexes, and in 2003 and 2004, its proportion was small or null.

### Temperature simulation experiment

We here tested whether temperature dynamics recorded in Fukaura in summer could induce modifications in a laboratory. We obtained 32 successfully eclosed individuals from Cycle A (see Methods), which produced three modified individuals in total (one outward type, one inward type, and one reduction type). They were all just slightly modified and not comparable with the field-caught modified Fukaura individuals in their degrees of modifications (not shown). From Cycle B (see Methods), we obtained 30 adults, but none of them were modified. These results indicate that temperature fluctuations during the pupal period in summer would not be an immediate cause for the outbreak of the modified forms in Fukaura.

### Artificial modifications: cold-shock experiment

The highest incidence of the modified forms in summer might at first suggest that heat shock in Fukaura induced modifications. We checked the past temperature records thoroughly but the highest temperature of Fukaura was not higher than those of southern parts in the mainland Japan. Indeed, *Z. maha *is mainly distributed in tropical or temperate area in Asia, and thus it should be resistant to "high" temperature in Fukaura. Furthermore, "high-temperature" treatment indeed produced no modification (not shown). Thus, despite the fact that the modified individuals were found during summer in Fukaura, we think that this is simply because of the increase of the entire population in summer.

On the other hand, the color-pattern modifications were indeed quite similar, if not identical, to the cold-shock-induced modifications previously reported in lycaenid butterflies [13,14]. At this point, we reasoned that northern expansion was made in response to warmer climate during summer, and *Z. maha *subsequently experienced cold stress in winter that had never been experienced before. We thus tested whether experimental application of cold shock could reproduce these color-pattern modifications. For this purpose, we first used pupae that were reared from eggs collected from normal females of *Z. maha *caught in Hiratsuka, a locality south of Fukaura (see Fig. 1A inset). The Hiratsuka population would have never been exposed to the environmental conditions experienced by the Fukaura population; nonetheless, the Hiratsuka population is expected to possess similar phenotypic plasticity. This possibility can be examined by a cold-shock treatment, as if the Hiratsuka population migrated to the north and encountered the colder environment.

**Figure 3:**
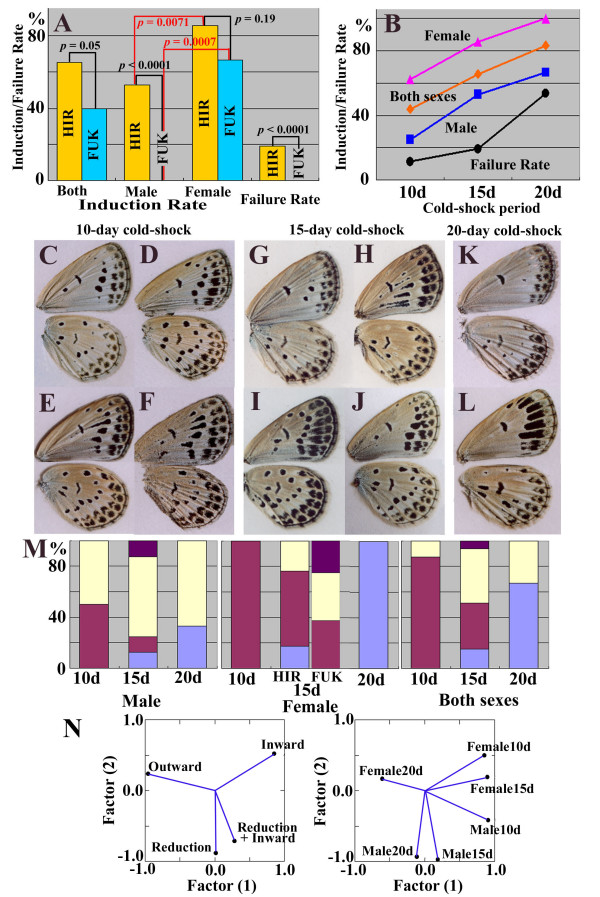
**Cold-shock experiment**. (A) Induction rate (IR) and failure rate (FR) of the Hiratsuka (HIR) and Fukaura (FUK) populations of *Z. maha *treated for 15 days at 4°C. Higher IRs in females and in the Hiratsuka population are observed. FR is also higher in the Hiratsuka population. The IR difference in male between the Hiratsuka and Fukaura population is conspicuous. (**B**) Dose-dependent responses of the IR (pink, orange, and blue lines) and FR (black line) of the Hiratsuka population. No modification was able to be clearly detected in 0-d and 5-d cold-shock treatments. ANCOVA indicated statistical difference in sexes (*p *< 0.001). (**C-L**) Various wing color-patterns of the cold-shock treated individuals for 10 days (**C-F**), 15 days (**G-J**), and 20 days (**K, L**). (**M**) Modification-type profiles of the cold-shock-treated *Z. maha *individuals. Result of the Fukaura individuals (FUK) for the 15 day treatment is also shown, but all other data were obtained from the Hiratsuka individuals (HIR). For the color assignment, see **Fig. 2S**. Note the step-wise increase of the outward type and decrease of the inward type, and almost constant proportion of the reduction type in male. (**N**) Principal component analysis, indicating relationships among the four modification types and between sexes.

We first performed a 15-day cold-treatment protocol using the Hiratsuka pupae (see Methods). We successfully obtained all three types (*i.e.*, the inward [Fig. 3H, I], reduction [Fig. 3G], and outward [Fig. 3J] types) of the modified individuals that were similar to the field-caught modified ones (Fig. 3), although the modifications found in these individuals were not as severe as those in the wild Fukaura individuals were. We obtained 36 modified individuals out of 55 that underwent successful eclosion, yielding an induction rate (IR) of 65% (Fig. 3A). Males and females showed different IRs; the male IR was 53%, whereas the female IR was 86% (*p *= 0.0071). We also defined the failure rate (FR) for an evaluation of the cold-shock resistance, and we obtained 13 "failed" individuals out of 68, yielding a FR of 19%.

We performed a similar experiment using pupae that were reared from eggs collected from normal females of *Z. maha *caught in Fukaura (Fig. 3A). We obtained 8 modified individuals out of 20 individuals that underwent successful eclosion, yielding an IR of 40%. No modified individuals were found among the 8 male individuals that were obtained (IR = 0%), whereas 8 modified individuals were obtained among 12 female individuals (IR = 67%). The difference between the male IRs of the Hiratsuka and Fukaura populations was statistically significant (*p *< 0.0001), although that of the female IRs scored *p *= 0.19. In the Fukaura population, the IR difference between males and females was notable (*p *= 0.0007). Furthermore, no individuals failed eclosion, *i.e.*, the FR was 0%. The difference of FRs between the Hiratsuka and Fukaura populations scored *p *< 0.0001.

### Dose-dependent response to the cold-shock treatment

We further treated the *Z. maha *individuals from Hiratsuka as above for different periods of cold application (10 days and 20 days in addition to the initial 15-day treatment) to observe a dose-dependent response to the cold treatment. Both IR and FR steadily increased from the 10-day to the 20-day application periods (Fig. 3B). The sex factor adjusted for the covariance cold-shock period was statistically significant in the mathematical model in which only sex and cold-shock period were predictors for IR (*p *< 0.001).

In the 10-day treated individuals, the inward type was mainly obtained (Fig. 3C-F, M). In the 15-day treatment, the proportion of the inward type became relatively small for both sexes (Fig. 3G-J, M), and in the 20-day treatment, the outward type dominated the population, especially in females (Fig. 3K, L, M). The experimental modification-type profiles (Fig. 3M) showed the sequential transition of the modification types with an almost constant proportion of the reduction type in males. That is, less severe temperature conditions (which correspond to the 10-day treatment) tended to produce the inward type. More severe conditions (which correspond to the 15-day treatment) tended to produce various types of modifications including reduction type. Very severe conditions (which correspond to the 20-day treatment) tended to produce the outward type. Principal component analysis clearly supported these relationships among the four different modification types and between sexes (Fig. 3N).

### Artificial selective breeding

We speculated that one of the driving causes for the Fukaura case is, in addition to the temperature stress during winter seasons, geographical isolation of the northernmost Fukaura population that allowed a founder effect for the modified forms (see Discussion). We performed artificial selective breeding of the outward type, which occupied more than 60% of the entire modified individuals in Fukaura, to see if this trait can be genetically fixed and, if so, how many generations are required for that process to occur.

**Figure 4:**
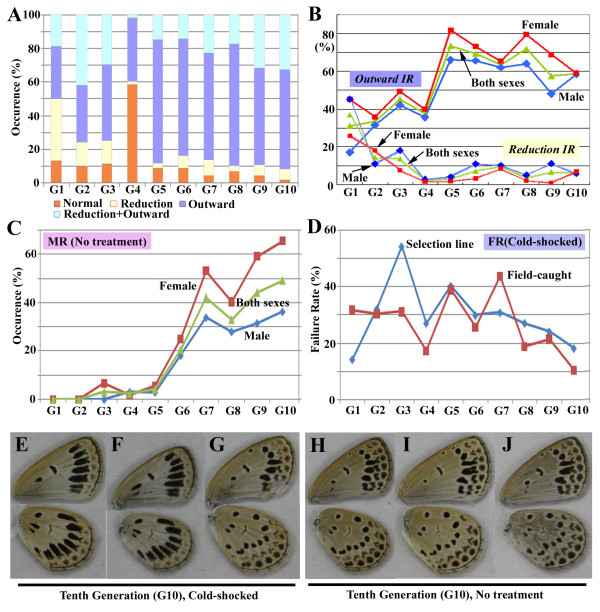
**Selective breeding experiment for the outward type**. (**A**) Modification-type profiles for generations (from the first generation [G1] to the tenth generation [G10]) after the cold-shock treatment. The fourth generation was treated differently, producing the smaller number of the modified individuals. The inward type was not produced. The outward type steadily increases until the fifth generation (G5). (**B**) The outward induction rate (IR) and reduction IR after the cold-shock treatment in males and females for generations. Females show higher outward IR than males do throughout generations. The outward IR appears to be in a plateau state after the fifth generation (G5). Males show higher reduction IR. (**C**) Modification rate (MR) for generations without immediate cold-shock treatment to pupae. As in the outward IR, females show higher MR than males do throughout generations. MR increases after the fifth generation (G5). (**D**) Failure rate (FR) after the cold-shock treatment for generations. FR of the selective line is less than 30% in G6 and in the following generations, indicating that the line is well maintained. The field-caught samples indicated by the red line were not genetically manipulated. Their parent females were caught freshly from the field to collect virgin adults for mating. (**E-G**) Modified individuals of the tenth generation (G10) in response to the cold-shock treatment. Both fore- and hindwings are severely modified as the outward type in many individuals. (**H-J**) Modified individuals of the tenth generation (G10) without immediate cold-shock treatment to pupae. Modifications are not severe but clearly observed.

Selection for the outward phenotype after cold-shock treatment steadily increased the proportion of the outward individuals up to the fifth generation (Fig. 4A). In the fifth generation, the outward type occupied 80% of the total population. The outward sensitivity to the treatment was increased fairly quickly despite the fact that selected individuals were crossed with field-caught individuals every generation. The proportion of the normal individuals decreased gradually to the tenth generation, indicating the feasibility of the selective breeding procedure. Most tenth generation cold-shocked individuals showed extensive outward modifications (Fig. 4E-G). Throughout the generations, the outward type comprised more females than males, as in the Fukaura case (Fig. 4B). Inversely, the reduction type comprised more males than females, also similar to the Fukaura case.

Intriguingly, we obtained a small number of the outward type without immediate cold-shock treatment to pupae in the third generation (Fig. 4C). From the sixth to tenth generations without immediate treatment to pupae, the proportion of the outward type (defined as modification rate, MR) suddenly increased to 30-50% and appeared to be further increasing up until the tenth generation, although the degree of modifications in a given individual was not very severe even in the tenth generation. Nonetheless, the outward modifications were clearly distinguishable from the normal color-pattern (Fig. 4H-J). Failure rate (FR) in the selection line became around 30% in the sixth generation and gradually decreased in the subsequent generations (Fig. 4D).

### Survey for historical records of the modified forms

No modified individual of *Z. maha *was described in the compilation of butterflies in Akita Prefecture [9,10]. However, two severe outward-type individuals caught in the previous northern rage margins of Akita Prefecture in September and October, 1989, were reported [15]. This is just two years before the northern expansion (Fig. 1A). However, modified individuals have been occasionally reported in the Japanese lepidopterological journals including the outward-type modifications (*e.g.*, [16]) as well as those of the inward and reduction types (*e.g.*, [17,18]) from many localities. We found 16 reports of the modified *Z. maha *individuals from 1987 to 2000 in two journals, which is about one report per year.

## Discussion

### Field record of the Fukaura outbreak: systematic modifications

In this paper, we reported the observation of the outbreak of the color-pattern modified individuals of *Z. maha *in its northern range margins, Fukaura, Japan. It is important to note that these modified individuals are, as far as we can tell, anatomically and behaviorally normal (except for the wing color-patterns), and the color-pattern modifications are thus essentially different from nonfunctional deformation or teratology caused by, for example, environmental pollutants. The observed systematic color-pattern modifications in the Fukaura individuals are indeed different from a general stress response [19,20].

To understand the importance of the Fukaura case, we ask how frequently the modified types occur in the field. Only one severe and two slightly modified forms were obtained among the collection of about 3,000 *Z. maha *individuals over many years in Kamakura, Kanagawa Prefecture, indicating that the occurrence of the modified forms in Kamakura (and probably elsewhere in Japan) is on the order of 0.1%. This seems to be consistent with the sporadic reports of the modified forms in the Japanese lepidopterological journals. On the other hand, this number is too large for a random genetic mutation. All modified forms reported in lepidopterological journals are similar to one of the three modification types found in Fukaura, which is also difficult to explain by a random mutation. Similarities in phenotypes between the Fukaura and experimental individuals are not easily explained by a random mutation, either. Most of the modified individuals reported in lepidopterological journals probably resulted from phenotypic plasticity. That is, it is likely that these individuals required cold shock to express the color-pattern changes without any genetic process.

In the Fukaura case, the color-pattern changes had been fixed genetically, because the modified individuals were observed in summer without cold shock. Since the Fukaura's modification occurrence was about 15% in the highest year, it is reasonable to think that the individuals modified both phenotypically and genetically became founders for the Fukaura population. Only in this way, a rapid expansion of the modified population would be attainable, judging from our genetic experiment. Thus, the most plausible scenario would be that founding individuals with the modified phenotypes and genotypes (but not produced by a random mutation) migrated to the Fukaura area and established the temporal population there (see below).

### Implications of the cold-shock experiment in the Fukaura outbreak

We here experimentally reproduced the color-pattern changes found in Fukaura by cold shock. The modification-type profiles differed by sex in the cold-shock experiment, which was also observed in the Fukaura individuals. Both in the modified Fukaura individuals and in the cold-shocked Hiratsuka individuals, the reduction type was male-preferred and less affected by temperature stress, and the outward type was female-preferred. The inward and outward types seem to constitute a continuous response series, and the outward type was more severe than the inward type. Indeed, the inward and outward modifications never coexisted in a single individual. Thus, females may be more vulnerable to the cold-shock induction of modifications. The increase of the outward type in males from 2002 to 2004 in Fukaura likely indicates ongoing process of genetic assimilation of this modification trait in the population.

Differences of phenotypic plasticity in response to experimental conditions were found between the Fukaura and Hiratsuka populations in the cold-shock experiments. Judging from the FRs, the *Z. maha *population of Fukaura in 2003 appeared to be more resistant to the cold-shock treatment than that of Hiratsuka, although this statement suffers from a small number of treated individuals. Furthermore, judging from the IRs, the color pattern of the Fukaura population in 2003 appeared to be more canalized against the cold-shock treatment than that of the Hiratsuka population, especially in male individuals. That is, it appears that the cold-shock resistance might have evolved especially in males of the Fukaura population. The possible resistance and canalization of the Fukaura population suggest that the individuals that could resist the cold shock efficiently and retain their normal color-pattern might have been preferably selected within the Fukaura population, implying the existence of a selective pressure for canalization of the normal color-pattern and against phenotypic diversification.

In different closely-related species of lycaenid butterflies, *Lycaenides idas *and *L. melissa*, slight differences in spot patterns was reported to play a role in mate discrimination [21]. This may not occur in *Z. maha*, according to previous behavioral experiments on this species [22-24]. However, a possibility that sexual difference in response to the cold-shock treatment may originate from sexual selection in mating behavior cannot be excluded at this point. Detailed behavioral experiments are necessary to settle this issue.

### Possible selection in the previous range margins

Although the modified phenotypes found in Fukaura were reproduced by physiological treatment, such a revelation of plastic phenotypes cannot solely explain the Fukaura outbreak, because we failed to induce the modifications sufficiently in the temperature simulation experiment. In fact, most modified individuals were found in September and October when temperature is high in Fukaura, indicating that the modified phenotypes induced in winter should be genetically fixed to achieve the outbreak in summer. One possibility is that natural selection promoted cold resistance that increases survival in the previous northern range margins, and this physiological trait was linked or associated with the color-pattern changes as a side effect. The modified forms may be naturally selected against in the previous range margins if disadvantageous behaviorally, but this selection may be weakened by habitat changes or a founder effect in Fukaura, leading to the outbreak of the modified forms. Similar evolutionary history has already been postulated in speciation of *Vanessa *butterflies [25-28]. This is an important point to understand what happened in Fukaura. Therefore, the plausible association between cold resistance and color-pattern changes should be experimentally demonstrated in the future.

It is important to note that the outbreak occurred at the third year of arrival at Fukaura, probably within about ten generations. We performed artificial selection for the outward phenotype for ten generations, and yet we were unable to obtain the severe outward type despite obtaining the outward type with relatively small degree of modifications. More generations may be necessary to achieve the severe outward individuals that are comparable to the Fukaura individuals. The rapid increase of the modified individuals from 2000 to 2002 immediately after the colonization of Fukaura cannot be achieved without a certain degree of previous genetic changes.

### Possible founder effect behind the Fukaura outbreak

In summer, it is generally hot in Japan including the range margin area, so that butterflies can fly north. But in winter, most of their offspring cannot withstand cold temperature. This cycle may select for cold-tolerant individuals at range margins. We think that in Japan *Z. maha *has expanded its range to the north in this way. Thus, in the populations of the previous range margins (*i.e.*, Hachiryu, Akita, Honjo, and Kisakata, but not Fukaura), the cold resistant characters had been genetically accumulated already at least to some extent, and some individuals had become genetically unstable in color patterns, as indicated by our historical survey. A small number of these unstable individuals, together with some normal individuals, probably migrated to the Fukaura area by chance, which caused a founder effect, leading to the rapid expansion and subsequent decrease of the modified individuals. Note that the founding individuals are not random mutants, and this is different from the conventional scheme of founder establishment associated with random genetic drift.

It is likely that the population of *Z. maha *in Fukaura is reasonably isolated from other populations of the same species because *Z. maha *individuals are quite small and do not fly over long distances. High mountains called Shirakami-sanchi surround Fukaura and only a narrow path is available along the seashore for the northern range expansion of small insects.

### Unstable population structure in Fukaura

The sudden drop of the proportion of modified individuals in 2005 was accompanied by the drop of the entire population size. Because of the founder effect in an isolated population, sibling crosses might have occurred often, which could have at least partly contributed to the eventual deterioration of the population. We know that asymmetrical modifications are often accompanied with the wing deformation or growth retardation of scales in a laboratory breeding as a result of sibling crosses [29]. Asymmetrically modified individuals found in Fukaura could indicate the genetic deterioration of the Fukaura population. General increase of the outward type from 2000 to 2004 in males may also support this possibility. Additional possibility is a failure of the mate recognition system due to the color-pattern changes.

We speculate that population structure was genetically and phenotypically in a non-equilibrium state in the Fukaura area from 2000 to 2005. This unstable non-equilibrium state seen before 2005 might have been going back to an equilibrium state after 2005 where the modified forms cannot exist. The population of this species itself may not be able to exist in the equilibrium state in the range margin habitat. In fact, in 2005, the entire population seems to be nearly extinct, although not completely.

### Role of phenotypic plasticity in the color-pattern modifications

Phenotypic plasticity can be the basis of physiological and evolutionary adaptation of organisms as seen in seasonal polyphenism of many butterflies [30-34]. In a given species, phenotypic plasticity is usually buffered by the stereotyped action of a hormone and its receptor [30,33] or by the capacitative function of heat-shock proteins [35,36]. Accordingly, the phenotypic plasticity of a species can theoretically be revealed in response to unexpected environmental changes, such as range expansion.

We have previously speculated that the cold-shock-sensitive molecular pathway might have played a role in the phenotypic diversification and speciation of some nymphalid and lycaenid butterflies when an ancestral species invaded a new environment where temperature fluctuation was relatively high [13,19,25-28]. The present case of *Z. maha *in Fukaura may be considered to have a similar scenario. Further elucidation of the putative cold-shock hormone [37,38] is expected to clarify the whole physiological and evolutionary picture of temperature-induced color-pattern changes in butterflies.

## Conclusions

We recorded the natural outbreak of modified phenotypes of a lycaenid butterfly, *Z. maha*, in Fukaura, Japan. Our experimental results suggest that genetically unstable founders from the previous range margin population and their establishment of the temporal population in the recent range margins are likely to have contributed to the outbreak of the modified phenotypes. The outbreak was supported by the natural revelation of plastic phenotypes in response to temperature stress in the previous range margins and possibly in the recent range margins as well. This case presents not only an evolutionary role of phenotypic plasticity in the field but also a novel evolutionary aspect of range expansion at the species level.

## Methods

### Butterflies

A lycaenid butterfly *Z. maha *is a small Asian butterfly with the wingspan about 25 mm. It has characteristic black spots on the ventral side of the fore- and hindwings. No significant color-pattern differences between sexes on the ventral side of the wings are known, but sex can easily be identified by the dorsal colors of the wings; male has bluish scales, and female has black scales. The simple and consistent spot pattern on the wings among the population of this species makes it easy to distinguish each spot and to examine their modifications, if any, among individuals and between sexes.

The natural history of *Z. maha *has been studied thoroughly by lepidopterists [11]. Briefly, in the warm east coast area of Japan facing the Pacific Ocean, which includes the Hiratsuka area, adults are observed from late March to early December, with adults emerging five to six times a year (in other words, *Z. maha *is multivoltine). In the range margins including Fukaura, adults appear to emerge three to four times a year. This species overwinters mostly as third and fourth (final) instar larvae, but many of these larvae die in winter due to cold temperatures in cold areas. Pupation and molting are problematic below 12°C and no pupation can be observed after the end of November, even in the warm east coast area of Japan. Pupal periods last about 7 days in summer and 14 to 20 days in spring. Pupation occurs on the undersurface of leaves or on small stones and fallen leaves. Its host plant, *Oxalis corniculata*, favors mown or abandoned open fields or rocky ground. Due to the diminutive size of this plant (*i.e.*, its height is less than 10 cm above the ground in most cases), it is highly likely that a significant percentage of pupae are found on rocky surfaces where temperature fluctuation is relatively high. The number of adult individuals in spring is small and the population peaks in summer.

### Fieldwork

Fieldwork was carried out in Fukaura (latitude 40°38.7'*N *and longitude 139°55.9'*E*) in the Aomori Prefecture, the northernmost part of Honshu Mainland, Japan. We surveyed nine locations in the Fukaura area (Iriaizaki, Henashizaki, Tsubakiyama, Sawabe, Moriyamazaki, Moriyama, Kurosaki, Itagai, and Iwadate). The number of sampling days was as follows: 6 (2000), 25 (May to October, 2001), 25 (2002), 20 (2003), 20 (2004), and 21 (2005). Sampling was carried out from the beginning of May to the end of November except 2001 (May to October but not in November) and 2000 (only in October). We carried out fieldwork every weekend or every other weekend from August to October, so that sampling days were mostly evenly distributed over the flight season (see Fig. 1D).

We estimated the percent of modified individuals in the Fukaura population for each year. We kept diary records in the field on how many individuals were normal or modified. Individuals were caught in nets, and most were released after checking the color-pattern normality or abnormality. We cannot exclude the possibility that the same individuals were counted twice or more. However, since catching an adult butterfly is more or less random, this redundant capture does not significantly influence the final percentage of the modified forms in the entire population. The relative population size for a year was estimated by assuming that we spent a similar amount of time performing fieldwork each day. Thus, the total number of identified individuals caught in a year was divided by the number of days of fieldwork in a year. The population size of the year 2000 was set at 1.0 and the population sizes of other years were normalized accordingly. For comparison, *Z. maha *individuals were caught in Hiratsuka (latitude 35°20.7'*N *and longitude 139°18.2'*E*) in Kanagawa Prefecture, Japan, whose longitude is similar to that of Fukaura. Both Hiratsuka and Fukaura individuals were used for the cold-shock experiments. Weather data were obtained from the weather stations operated by the Japan Meteorological Agency (Tokyo, Japan).

### Temperature simulation experiment

We looked for the temperature records of Fukaura from August 25 to September 7, 2003, since many modified individuals were found following this period. During this period, temperature fluctuated from 13.5°C to 27.9°C. Considering the possible microenvironment close to the ground that pupae could experience, we subjected pupae to two temperature cycles, Cycles A and B (see below). Temperature ranges were set to completely cover the lowest and highest temperatures. Larvae were reared as in the 4°C cold-shock experiment, and pupae were transferred to the programmable incubator FMU-053I (Fukushima Industries, Osaka, Japan). We set two temperature cycles as follows. Cycle A was carried out in the following order: 4°C (6 hours), 17°C (6 hours), 30°C (6 hours), and 17°C (6 hour). Cycle B was in the following order: 4°C (12 hours) and 30°C (12 hours). These cycles were repeated until eclosion.

### Cold-shock experiment without breeding

We both collected larvae in the field and obtained larvae from eggs laid by field-caught females from Hiratsuka. Larvae were reared at 25 ± 1°C with a 16L-8D photoperiod cycle. Within 10 hours after pupation, pupae were quickly transferred to and incubated at 4 ± 1°C for 10, 15, or 20 days and then returned back to the previous conditions. Larvae from Fukaura, hatched from the eggs collected from normal females caught in 2003, were similarly treated. So that the treated individuals would represent their regional populations, we used several female individuals from the fields, and the emerged adults were not allowed to reproduce. No modified individuals were obtained without the cold-shock treatment (*n *> 100). For quantitative analyses of the experimental results, induction rate (IR) and failure rate (FR) were defined as follows: IR(%) = {(the number of modified individuals)/(the number of successfully eclosed individuals)} × 100; FR(%) = {(the number of individuals that failed eclosion due to pupal death, wing deformation, or other unknown reasons plus the number of individuals that eclosed with severely deformed wings)/(the total number of individuals examined)} × 100. Because the adult individuals with severely deformed wings cannot survive after eclosion, FR is equivalent to the pupal mortality rate. Statistical significance was examined with a two-sided unpaired *t*-test using JSTAT 10.0 (2006) for Fig. 3A and with analysis of covariance (ANCOVA) using SYSTAT 13 (2009) for Fig. 3B. Before performing ANCOVA, homogeneity of slopes was checked by a model with the possible interaction between sex and cold-shock period (covariate). The probability value for sex by covariate interaction was 0.878, justifying the assumption of homogeneity of slopes. Principal component analysis was performed using SYSTAT 13 (2009). Occurrence variables were reduced to two components from Pearson correlation matrices with default varimax rotation and eigenvalues to maximize percentage of the total variances explained. Scree plots were checked for feasibility.

### Artificial selective breeding

The artificial selective breeding experiment was performed to reproduce the Fukaura case in a laboratory and has at least two purposes. First, it would be necessary to examine if the phenotypically plastic trait of wing color-patterns can be fixed genetically in a small number of generations. Second, this breeding experiment was designed to make a rough comparison to the field case possible. We crossed the breeding line with individuals from outside in each step. This is because even selected individuals would mate with non-selected ones in high frequency. This is indeed highly likely in the field, considering that the sibling crosses were found to be mostly fatal [29].

In this experimental system, if we can obtain the individuals that are modified as severely as the ones in Fukaura in about ten generations (This is because *Z. maha *reached Fukaura in 2000 and two years later, we observed the outbreak of the modified forms. This would take about ten generations, because three to four generations per year are seen in Fukaura.), we cannot exclude the possibility that the selection was carried out only in Fukaura without a founder effect. In contrast, if we obtain the individuals that are much less severely modified even after the tenth generation, it may suggest natural selection in the previous range margins and a founder effect upon the range margin expansion in Fukaura.

For the artificial selective breeding, we used individuals caught on the Nishihara Campus of the University of the Ryukyus in Okinawa, Japan. Detailed methods for rearing *Z. maha *for generations in a laboratory will be published elsewhere [29]. To begin the breeding line, we set up two separate lines, each of which originated from an adult female caught from the field. From these females, eggs were collected and hatched larvae were reared at ambient temperature (around 25°C) with a 16L-8D photoperiod cycle. Pupae were cold-shocked under various temperatures and durations as a pilot experiment. Eclosed virgin adults with relatively severe outward modifications from each group were selected and kept in a cage for mating, and the resulting eggs were collected. The adults obtained from these eggs were defined as the first generation. Larvae of the first and subsequent generations were reared at ambient temperature (around 25°C) with a 16L-8D photoperiod cycle.

Six to twelve hours after pupation (except for the fourth generation), pupae were transferred to and kept at -2 ± 1°C for 3 days and then returned back to the previous conditions. Again, among the successfully eclosed adults of the first generation, a few virgin individuals with the most severely modified color-patterns were selected for mating. For their mating partners, we set another batch of eggs collected from field-caught females in advance, which were reared and then cold-shocked at the pupal stage in the same way. Among the successfully eclosed adults, severely modified virgin individuals were selected for mating with the first generation line. This way, mating partners for each generation of the line were produced from the fresh field-caught females each time.

The cycle of egg collecting, larval rearing, pupal cold shock, outward-type selection, and mating was repeated until the tenth generation. For the fourth generation, pupae were cold-shocked at 6-24 hours after pupation due to our process error. The numbers of cold-shocked and successfully eclosed individuals for each generation, from the first to tenth generations, were as follows: 106, 170, 341, 768, 369, 307, 605, 406, 231, and 289. A group of pupae from each generation were not cold-shocked so that the genetic effects of the selection process could be examined. The number of modified individuals without immediate cold-shock treatment to pupae was counted and expressed as a percentage, which is called the modification rate (MR) in this paper. The numbers of individuals without immediate treatment that were used for the MR evaluation, from the first to tenth generation, were as follows: 94, 153, 193, 125, 71, 53, 90, 139, 106, and 154. FR was calculated for every generation as in the cold-shocked populations.

### Historical literature survey

We surveyed the historical records making reference to all issues of the two leading Japanese lepidopterological journals *Chouken Fields *from 1986 and *Butterflies *from 1992 for the modified phenotypes caught in the field as of January 2009. These two journals publish many records of field-caught aberrant forms of butterflies. We also referred to two local publications on butterflies in Akita Prefecture [9,10], which was the previous northern range margins of *Z. maha*.

## Authors' contributions

JMO contributed to the conception and design of the experiments, conducted the experiments and fieldwork, analyzed the data, and wrote the manuscript. AK conducted the experiments and analyzed the data together with MI. TK conducted the fieldwork and analyzed the data. All authors read and approved the final manuscript.
